# Do investments in human and physical capital respond differently to financing constraints?

**DOI:** 10.1186/s41937-022-00090-8

**Published:** 2022-05-01

**Authors:** Giorgio Brunello, Áron Gereben, Désirée Rückert, Christoph Weiss, Patricia Wruuck

**Affiliations:** 1 via del Santo 33, 35123 Padova, Italy; 2 European Investment Bank, 98-100 Boulevard Konrad Adenauer, 2950 Luxembourg, Luxembourg

**Keywords:** Training, Financing constraints, Europe, D22, D25, J24

## Abstract

Using a representative sample of European firms, we study whether financing constraints affect employers’ investments in employee training and physical capital differently. We measure financing constraints with an index that combines survey and balance sheet data. We instrument this index with the non-performing loans ratio of the bank that provided the last loan to the firms or with the average ratio of banks in the local area. We find that financing constraints have no effect on investment in training, but substantially reduce investment in physical capital.

## Introduction

About one in five companies in the EU report to have invested too little in the training of their workforce in 2017 (EIB, 2018). This is a source of concern because, in an economic environment characterized by globalization, population ageing and technological progress, it is necessary to constantly update the skills of workers, and firms have a key role in promoting lifelong learning.

Firms tend to under-invest into skills of their staff due to factors affecting the expected benefits and costs of training. The former comprise hold-up problems, employee poaching and high staff turnover,^1^ and the latter include financing constraints. When capital markets are not perfect, firms may not be able to invest as much as planned because they have difficulties in accessing external funds or because these funds are excessively costly. The costs of raising external finance are typically higher for firms with high leverage and low internal funds.

**Fig. 1:**
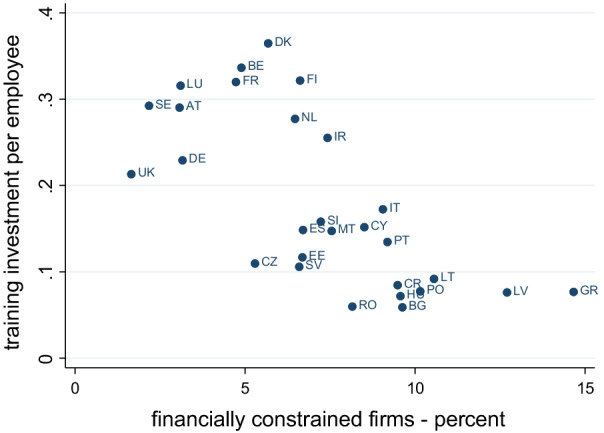
Investment in training per employee (TI) and share of financially constrained firms (AC), by country. EIBIS 2015–18. *Note:* training investment is in thousand euro. Firms are weighted with value added weights provided by EIBIS to obtain values that are representative of the business population. Number of observations: 33,528. AT: Austria; BE: Belgium; BG: Bulgaria; CY: Cyprus; CR: Croatia; CZ: Czech Republic; DE: Germany; DK: Denmark; EE: Estonia; FI: Finland; FR: France; GR: Greece; CR: Croatia; HU: Hungary; IE: Ireland; IT: Italy; LT: Lithuania; LU: Luxembourg; LV: Latvia; MT: Malta; NL: Netherlands; PL: Poland; PT: Portugal; RO: Romania; SE: Sweden; SI: Slovenia; SV: Slovakia; ES: Spain; UK: United Kingdom

The relationship between (self-reported) financing constraints and investment in training in Europe is shown in Fig. 1, which displays the country-specific share of firms reporting that they are financially constrained (on the horizontal axis) and investment in training per employee (in thousand euro on the vertical axis) for the 27 EU Member States and the UK during 2015–2018. Since the negative correlation could be driven by country-specific confounding factors, correlation does not imply that stronger financing constraints cause lower investment in employee training.

While there is a large empirical literature showing that financing constraints negatively affect investment in physical capital (see the surveys by Fazzari et al., 1988, and Hubbard, 1998), less has been done to investigate the effects of these constraints on employers’ investment in employee training. In the only study we are aware of, Popov (2014), uses cross-sectional data of the 2005 World Bank Enterprise Survey on the self-reported financing constraints of 8,265 small and medium-sized enterprises in 25 transition economies and finds that lack of access to finance in general, and to bank credit in particular, is associated with a significantly lower probability that the firm runs a formal on-the-job training program for its skilled employees. As the author admits, however, the measure of training he uses is somewhat coarse, as it treats as observationally equivalent a single short training course and a large-scale ongoing training program. In addition, his study needs to exclude firms with no skilled employees.

We contribute to this literature in two directions. First, we do not rely exclusively on self-reported constraints, but develop an index that combines these constraints with two financial indicators–leverage and the cash flow to assets ratio–from the balance sheets of firms. This index is based on the idea that self-reported constraints in survey data are more credible when they are backed up by hard financial data (see Lamont et al., 2001 and Hadlock & Pierce, 2010, for a similar approach). We show that the estimated marginal effect of financing constraints on training investment changes sign when these constraints include balance sheet information.

Second, while Popov focuses on firms with skilled employees in small and medium-sized firms in developing economies and on the incidence of training, we consider all the employees of firms with at least five workers in developed economies and use data on investment in employee training, which varies with training intensity, costs and duration and includes both formal and not formal training.

Our data are drawn from the EIB Investment Survey (EIBIS), which includes information on both self-reported financing constraints and investment in training and physical capital. EIBIS data are matched at the firm-level to balance sheet information from the Bureau van Dijk’s Orbis database. EIBIS also asks firms for the name of the bank that provided the last bank loan, which allows us to use information on banks’ financial ratios from Bureau Orbis Bank Focus (Bureau van Dijk).

Our sample of firms cover all the 27 EU Member States and the UK. These countries differ in their systems of financial intermediation in spite of the regulatory framework that has become more harmonised over time. EIBIS data are collected in a consistent manner and with the same methodology for a large number of firms across different countries, making it possible to carry out a comparative analysis of financing constraints and investment in employee training in diverse institutional settings.

We recognize that estimating the effect of financing constraints on investment in training and physical capital is complicated by the presence of unobserved heterogeneity, reverse causality (running from investment to financing constraints) and measurement error. We address these problems by instrumenting endogenous financing constraints with the non-performing loans ratio of the bank that provided the last loan or, when this is not available, with the average ratio for banks in the local area.

Our identification relies on the argument that, conditional on aggregate and firm-specific demand factors, financing constraints are driven also by supply factors, such as the tighter credit standards used by banks, which are affected by the expected profitability of their existing loan portfolio. We use the non-performing loans ratio as an indicator of supply-side constraints. Credit standards influence employer-provided training only indirectly, by altering financing constraints.

We estimate the causal effect of financing constraints on per capita investment in both human and physical capital. In contrast to Popov (2014), who finds that self-reported constraints reduce both training and physical capital investment, our results indicate that a one standard deviation increase in the financing constraints index has no effect on investment in training, but a sizeable negative (− 53 percent) effect on investment in physical capital.

These results suggest that the presence of financing constraints is unlikely to be a factor explaining the perceived under-investment in training in Europe. They also indicate that, since both the investment in training and in physical capital affect the productivity of firms, a mechanism whereby financing constraints influence productivity is that they significantly alter the accumulation of physical capital, but have no relevant effect on human capital.

The remainder of this paper is organised as follows. Section 2 briefly reviews the relevant literature. Section 3 presents the data and Sect. 4 introduces the financing constraints index. Ordinary least squares (OLS) and instrumental variables (IV) estimates of the effects of financing constraints on investment in human and physical capital are reported in sects. 5 and 6. Section 7 considers the effects of human and physical capital investment on firm productivity. Section 8 discusses robustness exercises. Conclusions follow.

## Review of the literature

When training is entirely ‘general’ and the labour market is perfectly competitive, we know from Becker (1964), that the worker should pay for it. In this case, the financial constraints that matter are those faced by workers rather than by firms. However, when training is entirely or partly firm-specific, or is general but the labour market is imperfectly competitive, the firm is willing to share or pay the costs (Acemoglu & Pischke, 1999; Hashimoto, 1981). In these circumstances, the financing constraints faced by firms can affect the provision of training.

In a world of frictionless financial intermediation, a firm’s financial structure does not affect its market value and firms’ decisions, motivated by the maximization of shareholders’ claims, are independent of financial factors (Modigliani & Miller, 1958). However, there are a number of reasons why financial intermediation is not frictionless. These include taxes, transaction costs and information asymmetries between lenders and borrowers and/or between managers and shareholders, which make external sources of finance more expensive than internal finance.

When markets are characterised by information asymmetries, external finance is available only on less favourable terms in capital markets, or is not available at all. Under such circumstances, investment spending is constrained by the shortage of internal funds, or cash flows (Fazzari et al., 1988), and credit rationing may occur.^2^ Any investment activity can potentially be adversely affected by a rise in borrowing costs – including investment in employment (Nickell & Nicolitsas, 1999; Boeri et al., 2018; Breunig et al., 2020) and research and development (Brown et al., 2012). By affecting investment, credit rationing can also have an impact on firm productivity (Ferrando & Ruggieri, 2018).^3^

There is ample empirical literature examining the effect of financing constraints on capital expenditure. In a seminal contribution, Fazzari et al. (1988), found evidence of a positive association between investment and the ratio of cash flow to capital—used as a proxy of financing constraints–after controlling for Tobin’s Q. However, since the cash flow ratio could also correlate with the profitability of investment that is not captured by Tobin’s Q, the uncovered association is likely to be biased (see Campbell et al., 2012).

An alternative measure of financing constraints is the Kaplan–Zingales (KZ) index (see Farre-Mensa & Ljungqvist, 2016), a linear combination of five readily available accounting variables: cash flow to total capital, market-to-book ratio, leverage (debt to total capital), dividends to total capital, and cash holdings to total capital. Lamont et al. (2001), compute this index using as weights the estimated coefficients from the regression of the qualitative measure of financing constraints developed by Kaplan and Zingales (1997),^4^ on the five accounting variables.^5^

Another measure of financing constraints used in the literature relies upon the replies by firms to direct questions asking whether they were denied credit, or did not apply for it in the first place, fearing that they would be rejected. Studies using self-reported constraints include (Beck et al., 2005; Campello et al., 2010; Popov, 2014; and Ferrando & Mulier, 2015). In a recent paper, Garcia-Posada Gomez (2019, uses data from a large panel of small and medium-sized enterprises in 12 European countries for the period 2014–2016 and finds that self-reported credit constraints, both in bank and other financing (e.g. trade credit), have strong negative effects on investment in fixed assets.

In the only empirical paper investigating the effects of (self-reported) financing constraints on training, Popov (2014), estimates that, all else equal, a credit constrained firm has as much as a 9.3 percent lower probability of running a formal on-the-job training program for its skilled employees than a firm which is not constrained in credit markets. He also finds that this effect is stronger in industries that employ a more skilled workforce and face good global growth opportunities.

## The data

We use firm-level data on investment in training and self-reported financing constraints from the EIB Investment Survey (EIBIS), which cover the financial years 2015 to 2018. The EIBIS is administered each year to the senior managers or financial directors of a representative sample of firms in each of the 27 EU Member States and in the UK. The survey is run on a stratified sample of firms with at least five employees, with both full-time and part-time employees being counted as one employee, and employees working less than twelve hours per week being excluded.^6^

EIBIS data are matched to balance sheet information from the Bureau van Dijk’s Orbis database.^7^ The financial information in Orbis originates from business registers collected by local chambers of commerce to fulfil legal and administrative requirements, and is relayed to Bureau van Dijk via different information providers. Bureau van Dijk prepares the data from administrative sources and arranges them in a standard format to facilitate comparisons of business accounts across firms in different countries. EIBIS also asks to firms the name of the bank that provided the last bank loan, which allows us to use information on banks’ financial ratios from Bank Focus (Bureau van Dijk).

To reduce the influence of outliers, we drop for each relevant variable the observations above the 99 percentile.^8^ Our working sample with non-missing observations on training investment per employee consists of 8,780 firms in financial year 2018 and 35,808 firm-year observations over the period 2015–2018. About half of the sample (18,669 observations) is made of firms with more than one observation over the sample period.

While EIBIS data are complete for all firms in the sample, many relevant financial and economic variables drawn from Orbis have missing values. For instance, leverage and cash flow have 20,958 and 18,467 observations respectively, significantly less than the 35,808 observations in our working sample. To replace missing values, we use the average values in the same country, firm size class and sector (two-digit NACE classification) and for each variable we define an indicator variable equal to 1 when observations are missing, and to 0 otherwise.^9^ We evaluate in Sect. 7 whether our estimates are sensitive to the omission of imputed values and find that the results are similar, although somewhat less precise.

In EIBIS, the information on investment in training is based on the responses to a question that asks how much the business invested—in the relevant financial year—in the training of employees. We define training investment per employee TI as the ratio of investment in training in year *t* to the number of employees in the same year, deflated by the consumer price index.^10^

Focusing on 2015, we compare average training per employee TI in our sample with the average reported by the Continuing Vocational Training Survey (CVTS), an employer survey carried out by Eurostat every five years, including 2015, which also covers the EU27 Member States and the UK. The two surveys use different definitions of training: while EIBIS includes all the training the employer pays for, CVTS considers only planned training and excludes apprenticeships and firms with less than 10 employees.

In the EIBIS sample used in this study, average training per employee in 2015 was equal to 220 euro, less than half the value reported in the CVTS (585 euro) in the same year.^11^ Our data do not include information on the share of trained employees. According to the CVTS, this share was equal to 40.8 percent in 2015.^12^ Using this, we estimate that, in our data, average investment in training *per trained employee* in 2015 was 539 euro (or 220/0.408).

Table 1 shows that average training investment per employee during the period 2015–18 was equal to 227 euro, and Fig. 1 illustrates how this value varied across countries during the same period, showing that it was above 300 euro in Belgium, Denmark, Finland, France and Luxemburg, and below 100 euro in Latvia, Croatia, Greece, Lithuania, Hungary, Poland, Bulgaria and Romania.

**Table 1:** Descriptive statistics for the main variables 2015–2018

Variable	Mean	SD
Training investment per employee (thousand euro)	0.227	0.319
Investment in physical capital per employee (thousand euro)	6.248	9.586
Self-reported financial constraints *AC* (% of firms)	0.055	0.228
Index *FC* (financing constraints index)	0	1
Leverage: debt to assets ratio	0.532	0.199
Cash flow to assets ratio	0.103	0.060
NPL ratio (percent)	7.126	6.479
Return on equity	0.279	0.237
Real sales per capita	222.805	177.423
Firm age	4.586	0.779
Total assets (log)	4.001	2.099
Subsidiary (% of firms)	0.361	0.480
Foreign owned (% of firms)	0.225	0.418
Hampered by business regulations (% of firms)	0.287	0.452
Hampered by labour market regulations (% of firms)	0.284	0.451
Hampered by lack of staff with the right skills (% of firms)	0.457	0.498

Weighted means using EIBIS value added weights. Firm age: 1: less than 2 years; 2: 2 to 5 years; 3: 5 to 10 years; 4: 10 to 20 years; 5: more than 20 years

We also define investment in physical capital per employee KI by adding up investment in land, buildings and infrastructure, machinery and equipment and software, data and IT systems. As shown in Table 1, the sample average for this type of investment per employee during the sample period is 6,248 euro, which corresponds to 76.6 percent of total investment (compared to 9.78 percent for investment in employee training).

## The index of financing constraints FC

The relevant literature has measured financing constraints using either balance sheet or survey data. Some authors (see for instance Lamont et al., 2001; Hadlock & Pierce, 2010) have combined these two approaches, as we do in this paper.

EIBIS includes a measure of self-reported financial constraints (AC), which is based on the most recent loan application of the firm. It combines four indicators: (i) quantity constrained (the firm is unsatisfied with the amount of external finance obtained); (ii) rejected (the firm has seen its request for external financing rejected); (iii) price constrained (the firm decided not to seek any external financing because of excessive costs); (iv) discouraged (the firm decided not to seek any external financing due to the concern of being rejected). Each indicator is a binary variable equal to one if the firm reports a positive answer, and to zero otherwise. The binary variable AC is equal to one if any of these four indicators is non-zero, and to zero otherwise.^13^

As indicated by Table 1, the percentage of firms in our sample reporting to be financially constrained (AC = 1) is 5.5, but the value is lower in Western and Northern Europe than in Central, Eastern and Southern Europe. Figure 1 shows that it is highest in Greece (14.41 percent) and lowest in Sweden (2.33 percent). It is also higher among firms with 5 to 49 employees (7.6 percent) than among firms with more than 50 employees (4.5 percent), highest in the information and communication sector (7.9 percent) and lowest in the transportation and storage sector (3.8 percent).

A problem with self-reported financing constraints is that they may not reflect objectively the financial position of firms. For instance, less capable managers may report higher constraints—by claiming to have been rejected or discouraged from applying for funding – in an effort to shift the blame of inefficiency in their firm to the credit market (Popov, 2014).

We believe that a more reliable indicator of financial distress can be obtained by combining the data on self-reported constraints with information drawn from the financial statements of firms, and by recognizing that access to external finance is typically more problematic for firms with high leverage – measured as the debt to assets ratio—and low cash flow (see Lamont et al., 2001; Ferrando & Mulier, 2015).

On the one hand, firms with a higher leverage require higher profits to repay their debt, and are therefore more likely to default and have a lower credit rating (see Traczynski, 2017).^14^ On the other hand, a higher ratio of cash flow to assets indicates the availability of internal resources to fund expenditures without turning to external finance. Our data indicate that firms that report to be financially constrained have on average higher leverage (0.571 versus 0.530) and a lower cash flow ratio (0.089 versus 0.104) than other firms.

We could capture financing constraints by using self-reported constraints, leverage and cash flow as separate variables. Three endogenous variables, however, would require at least three instrumental variables for the identification of causal effects, which is a difficult task. A viable alternative that requires only one instrumental variable is to reduce the dimensionality of the problem by extracting a single variable from these three variables.

We use principal component analysis (PCA) to construct the index of financing constraints FC as the linear combination of three (standardized) variables: the variable AC, which measures self-reported financing constraints, leverage and the cash flow to assets ratio. We select the weights of the linear combination using the scoring coefficients associated with the first component of PCA, which corresponds to the only eigenvalue higher than one.

The index is given by

1 $$FC=0.670 std\left(Leverage\right)- 0.658 std(\frac{Cashflow}{AS})+0.344 std(AC)$$

where AS is for total assets and *std* is for a standardized variable. We notice that the weight assigned by PCA to the two balance sheet variables is almost twice as big as the weight assigned to self-reported information.

Using the value-added weights provided by EIBIS, the FC index has mean equal to 0.067 and standard deviation equal to 0.984. Negative values of the variable FC should thus be interpreted as lower than average financing constraints, and positive values as higher than average constraints. On average, FC is equal to − 0.115 for firms reporting no financial constraints (AC = 0) and to 1.296 for firms reporting these constraints (AC = 1). Therefore, a shift of the binary variable AC from zero (no reported constraints) to one (constrained) corresponds to a 1.181 standard deviations increase in the index FC.

Table 2 illustrates how the average value of FC differs for firms that report to be financially constrained from those that are not (AC = 1 or AC = 0), depending on whether they have leverage and the cash flow to assets ratio are above or below their sample means. For instance, depending on whether firms report to be financial constrained or not, the average FC decreases from 2.276 to 0.863 for firms with below-average cash flow and above-average leverage, and from 0.182 to − 0.964 for firms with below-average leverage and above-average cash flow ratio.

**Table 2:** Average values of the financing constraints index FC. By level of leverage, cash flow to assets ratio and self-reported financial constraints (AC). 2015–18

		Cash flow ratio > mean	Cash flow ratio $\le$ mean
Financially	Leverage > mean	1.379	2.276
constrained (self-reported)	Leverage $\le$ mean	0.182	1.202
Non financially	Leverage > mean	− 0.059	0.863
constrained (self-reported)	Leverage $\le$ mean	− 0.964	− 0.177

Weighted means using EIBIS value added weights

One implication of Eq. (1) is that, depending on whether they report to be financially constrained or not (AC = 1 or AC = 0), two firms with the same cash flow ratio share the same value of the FC index if their leverage is equal to 0.09 and 0.6 respectively. We treat the former firm as more exposed to financing constraints than the latter only if its leverage is higher than 0.09.

## OLS estimates

In this section, we estimate by ordinary least squares (OLS) the empirical relationship between investment per employee *Y* by firm *i* at time *t* and financing constraints *X*

2 $${Y}_{it}={{\beta }_{0}W}_{it}+{{\beta }_{1}X}_{it}+{u}_{it}$$

where *Y* is investment in training (TI) or physical capital (KI), *X* is either self-reported constraints AC or the index of financing constraints FC, *W* is a vector of control variables (which includes the constant term), *u* is the disturbance term and both AC and FC are standardized to have zero mean and unit standard deviation. The vector *W* includes the first and second lags of the return on equity–computed as the ratio of operating profits to shareholders’ funds—and the first and second lags of real sales per capita. These two variables capture firm-specific shifts to the marginal benefits of investment in training and physical capital.

We control for macroeconomic factors and aggregate and sectoral shifts to these marginal benefits with country by year and sector fixed effects. These effects also capture cross-country and cross-sector variations in the marginal costs of investment. Additional controls include firm size (measured by the log of real total assets), firm age,^15^ subsidiary and foreign ownership status, and binary variables for whether firms report to be hampered in their investment decisions by labour market regulations, business regulations, or the lack of staff with the right skills.^16^

In Sect. 3, we have argued at length in favour of a financing constraints index that incorporates both self-reported information and balance sheet data. We therefore start by comparing the effects on training investment of AC*,* FC and the balance sheet variables entering in FC*.* Column (1) in Table 3 reports our estimates of Eq. (2) with self-reported financing constraints AC; column (2) uses leverage; column (3) the cash flow to assets ratio; column (4) both leverage and cash flow; and column (5) the index FC. Each column of the table reports the estimated marginal effect, after conditioning on the variables in vector *W*.

**Table 3:** The effect of alternative indicators of financing constraints on investment in training per employee (TI). OLS estimates. 2015–18

Variable	(1)	(2)	(3)	(4)	(5)
Standardized AC	0.004**				
	(0.002)				
Standardized leverage		− 0.005**		− 0.004**	
		(0.002)		(0.002)	
Standardized cash flow			0.004**	0.004*	
			(0.002)	(0.002)	
FC					− 0.003*
					(0.002)
Sample size	28,494	28,494	28,494	28,494	28,494

All regressions include country by year and sector fixed effects, indicator variables for missing values, subsidiary and foreign status, for being hampered by business regulations, labour market regulations and lack of staff with the right skills, the first and second lag of return on equity and real sales per employee, firm age and the log of total real assets. Standard errors (in parentheses) are clustered by firm. *, **, *** for statistical significance at the 10, 5 and 1 level of confidence

Contrary to intuition, our estimates report a *positive* correlation between self-reported constraints AC and investment. On the other hand, firms with higher leverage and lower cash flow to assets ratios invest less, as one would expect. There is also evidence of a negative correlation between training investment and the index FC. These results suggest that combining self-reported and balance sheet information, as we do in this paper, produces different results than relying exclusively on self-reported data, as done by Popov (2014).

In Table 4, we use investment in training and physical capital as the dependent variable and report more in detail the OLS estimates of Eq. (2) using the index of financing constraints (FC).^17^ We find that FC has a negative effect on investment in training (TI) and physical capital (KI), but that the association with TI is less precisely estimated than the association with KI.

**Table 4:** The effect of the financing constraints index (FC) on investment in training per employee (TI) and in physical capital per employee (KI). OLS estimates. 2015–18

Variable	Training	Physical capital
FC (financing constraints index)	− 0.003*	− 0.532***
	(0.002)	(0.056)
Return on equity (t−1)	0.006	− 0.187
	(0.009)	(0.217)
Return on equity (t−2)	0.005	0.098
	(0.007)	(0.179)
Real sales per capita (t−1) × 1000	0.132***	3.870***
	(0.027)	(0.811)
Real sales per capita (t−2) × 1000	0.044	1.733***
	(0.029)	(0.864)
Subsidiary firm	0.019***	− 0.711***
	(0.005)	(0.159)
Foreign owned firm	0.016***	− 0.393***
	(0.006)	(0.160)
Log total real assets	0.003***	0.551***
	(0.001)	(0.130)
Firm age	− 0.004*	− 0.225***
	(0.002)	(0.069)
Hampered by business regulations	0.002	0.551***
	(0.004)	(0.130)
Hampered by labour market reg	0.007	0.020
	(0.004)	(0.125)
Hampered by lack of right skills	0.032***	0.311***
	(0.004)	(0.112)
Percent change due to one standard		
deviation change in FC	− 0.013*	− 0.083***
Sample size	28,494	27,553

All regressions include country by year and sector fixed effects and indicator variables for missing values. Standard errors (in parentheses) are clustered by firm. *, **, ***for statistical significance at the 10, 5 and 1 level of confidence. Percent change: estimated percent change in training per employee induced by a one standard deviation increase in *FC*

We estimate that a one standard deviation increase in the FC index reduces TI by 1.3 percent and KI by 8.3 percent. There is also evidence that training investment per employee increases with lagged real sales per capita, and tends to be higher for larger and younger firms, subsidiary and foreign owned firms, and firms that are hampered in their activity by lack of staff with the right skills. As suggested by Stevens (1994), such firms can try to attenuate hiring constraints by investing in additional training. Conditional on the positive and large effect of lagged real sales per capita, investment in physical capital is lower for subsidiary and foreign owned firms and tends to be larger for larger and older firms, and firms that are hampered by business regulations or by the lack of staff with the right skills.

Since there are several firms with zero investment, we also estimate Eq. (2) using a Tobit specification. The results are very similar to those reported in Table 4. In particular, we find that the effect of the index FC on training and capital investment is equal to − 0.004 (standard error: 0.003) and − 0.569 (standard error: 0.064) respectively.

## IV estimates

The ordinary least squares estimate of Eq. (2) are biased for several reasons. First, reverse causality may impart a positive bias if firms that invest less in training or in physical capital are also more liquid, less exposed to banks and therefore less financially constrained. Second, unobserved heterogeneity—including managerial ability—may affect both training and financing constraints, violating orthogonality conditions. Third, the index of financing constraints FC may contain measurement error, which attenuates OLS estimates. Because of these biases, the association between the FC index and investment in training or physical capital reported in Table 4 cannot be interpreted as a causal relationship. To identify a causal effect, we use instrumental variables.

Firms face financing constraints because of both demand and supply factors. While the former include aggregate, sectoral and firm-specific demand conditions, alternative sources of finance and the health of balance sheets (see Bending et al., 2014), the latter comprise banks’ funding conditions (availability of liquidity, funding costs), the opportunities for sharing lending risks (securitization) and banks’ risk-taking capacity (capital adequacy, non-performing loans).

We select our instrumental variable among the supply factors. Contrary to demand factors, which affect both financing constraints and the decision to invest in training or in physical capital, supply factors affect investment only indirectly, by changing financing constraints.^18^ We instrument these constraints with the NPL ratio, the ratio of non-performing loans to total loans of the bank that provided the last loan to the firm, or, when it is not available, with the average NPL ratio of banks in the local area.^19^

By affecting its risk-taking capacity, the NPL ratio influences the credit standards of the bank which receives the application. Bending et al. (2014), for instance, show that a one percentage point increase in the NPL ratio decreases net lending by around 0.8 percentage points. High non-performing loans reduce bank profits, by requiring higher provisions, lowering interest income, and generating higher expenses associated with their monitoring and management. The NPL ratio features higher risk weights, leading to higher capital needs. To maintain or boost capital adequacy, banks may deleverage, leading to a contraction in credit supply (see Huljak et al., 2020).

We construct the NPL ratio as follows: first, for close to 54 percent of the firms in 2018 and for about 27 percent of the firms in 2015 to 2017, we are able to identify the relevant bank, defined either as the bank providing the last loan or as the main bank (when more than one bank is involved). We assign to these firms the ratio of non-performing loans to total loans of the relevant bank, which we obtain from BankScope.

For the remaining firms that cannot be matched to a bank, we use the annual average NPL ratio in the local area (NUTS2 level), also from BankScope. In the very few cases when the local area of the firm is missing, we use the annual average level of NPL ratios provided by the ECB for each country. By combining firm-specific information with data that varies by area and year, we obtain an instrument that exhibits residual variation even after controlling for country-by-year and sector fixed effects.^20^ The sample average of the NPL ratio is 7.1 percent (standard error: 6.5), ranging from 37.4 in Greece to 2.8 in in Finland.

We present our IV estimates in Table 5. The first stage regression of the FC index on the instrument NPL plus the controls in vector *W* shows that the NPL ratio has a positive and statistically significant effect on FC (estimated coefficient: 0.006, standard error: 0.001). The F-test for the exclusion of the instrument ranges between 20.84 and 22.61, well above the standard rule of thumb (10), suggesting that the instrument is not weak.

**Table 5:** The effect of the financing constraints index (FC) on investment in training per employee (TI) and in physical capital per employee (KI). IV estimates. 2015–18

Variable	Training	Physical capital
FC (financing constraints index)	0.001	− 3.311**
	(0.053)	(1.683)
Return on equity (t−1)	0.006	− 0.187
	(0.009)	(0.245)
Return on equity (t−2)	0.001	0.405
	(0.010)	(0.357)
Real sales per capita (t−1) × 1000	0.132***	4.081***
	(0.029)	(0.857)
Real sales per capita (t−2) × 1000	0.048	2.019***
	(0.030)	(0.942)
Subsidiary firm	0.020***	− 0.905***
	(0.007)	(0.207)
Foreign owned firm	0.016***	− 0.522***
	(0.006)	(0.187)
Log real total assets	0.004***	0.844***
	(0.001)	(0.038)
Firm age	− 0.004	− 0.283***
	(0.003)	(0.081)
Hampered by business regulations	0.003	0.644***
	(0.004)	(0.152)
Hampered by labour market reg	0.007	0.152
	(0.005)	(0.107)
Hampered by lack of right skills	0.032***	0.353***
	(0.004)	(0.121)
*First stage*		
Non-performing loans ratio	0.006***	0.006***
	(0.001)	(0.001)
F-test	20.84	22.61
Percent change due to a one standard		
deviation change in FC	0.003	− 0.530***
Hansen J using Lewbel’s method – p-value	0.634	0.162
Sample size	28,494	27,553

All regressions include country by year and sector fixed effects and indicator variables for missing values. Standard errors (in parentheses) are clustered by firm. *, **, ***for statistical significance at the 10, 5 and 1 level of confidence. Percent change: estimated percent change in training per employee induced by a one standard deviation increase in *FC*

The IV estimates indicate that financing constraints do not alter the training investment (TI) but negatively affect the investment in physical capital (KI). We find that the coefficient associated with the FC index is very close to zero (0.001, standard error: 0.053) for TI and negative, large and statistically significant (coefficient: − 3.311, standard error: 1.683) for KI.

These estimates are local average treatment effects, or the effects for the sub-sample of firms that have their FC index altered by variations in the instrument (see for instance Angrist & Pischke, 2008). They imply that a one standard deviation increase in the tightness of financing constraints has no statistically significant effect on investment in human capital (training) but reduces investment in physical capital by 53 percent.^21^

When we break down investment in physical capital into its components, we find that the negative effect of the FC index is much larger for investment in land, business buildings and infrastructure (− 142.6 percent) than for investment in machinery and equipment (− 28.4 percent) and software, data and IT (− 37.3 percent).^22^

Recalling that a shift of self-reported constraints AC from 0 to 1 is close to a one standard deviation increase in the FC index, our estimate of the effect of FC on investment in physical capital is within the same ballpark but larger than the one reported by Popov (2014), who finds that a firm that needs a loan but does not have one because it is rejected or discouraged spends 40 percent less on capital investment.

The comparison of OLS and IV estimates suggests that, contrary to what happens for training investment (TI), the OLS estimate of the effect of the FC index on investment in physical capital (KI) contains a large positive bias, which could be due to shocks that contemporaneously reduce KI and the risk of being financially constrained, or to the fact that the impact of reverse causality–lower capital investment reducing the need to recur to external finance and therefore financing constraints—is much stronger for KI than for TI.

We verify whether the large difference in the estimated responses of TI and KI to changes in the FC index is statistically different from zero by pooling the data for the two types of investment and by estimating a specification which includes both the FC index and its interaction with a binary variable equal to 1 for physical capital and 0 for training.^23^ We find that the interaction term is equal to − 3.830 (standard error: 0.480), indicating that the null hypothesis of no differential response is rejected by our data.

The different responses of TI and KI to changes in the FC index may reflect the fact that training and physical capital are inherently different items, with training belonging to working capital (the difference between current assets and current liabilities), as wages and employment, while physical capital belong to investing capital, or the set of long-term fixed tangible assets of a firm. In support of this view, we find that changes in the FC index do not significantly affect average wages per employee and employment.^24^

Training investment could also be less responsive to changes in financing constraints than investment in physical capital because the provision of training within firms in Europe is often affected by the presence of CVT (continuous vocational training) agreements involving unions, which limit managerial discretion. According to the CVTS survey, in 2015 close to a quarter of firms had such agreements in place.^25^

Finally, financing constraints may have a stronger impact on physical capital investment than on training because the former often require larger budgets to undertake investment activities. Investments in physical capital are by far lumpier than human capital investments, which implies greater discontinuities in the former than in the latter in a context of financing constraints. A related aspect is that, even in a context of no financing constraints, the investment in employee training may already be too low, due for instance to the presence of poaching externalities. If the reference value of training investments is already too low, it is more likely that financing constraints do not matter.

Since our IV model is just-identified, we cannot test whether the excluded instrument (NPL) is appropriately independent of the error term. In order to perform a “Sargan-Hansen” test of the orthogonality conditions,^26^ we use Lewbel’s method (Lewbel, 2012) to generate additional instruments and obtain an over-identified model. In short, this method consists of: (a) obtaining the residuals of the first stage regression; (b) multiplying the demeaned exogenous regressors by these residuals and treat the products as additional instruments. When we do so, we find that the Hansen test fails to reject the null hypothesis that all instruments are orthogonal to the error term, as the p-value of the test is equal to 0.634 for TI and to 0.162 for KI, well above the five percent level of confidence.

## The effect of investment in training and in physical capital on productivity

A reason why we are interested in the relationship between financing constraints and investment in training or physical capital is that these investments could affect the productivity of firms. Financing constraints, by reducing investment, could be detrimental to firm performance. In this section, we investigate the relationship between investment in training and physical capital and productivity by estimating the following Cobb Douglas production function (see Bartel, 1991, and Mehra et al., 2014, for a similar specification) for firm *i* at time *t*^27^

3 $${Y}_{it}={A}_{it}{[{E}_{it}(1+\theta {TI}_{it})]}^{\alpha }{{K}_{it}}^{\delta }{{M}_{it}}^{\gamma }$$

where *Y* denotes output (sales, from Orbis), *E* the number of employees (from EIBIS), *K* the capital stock and *M* the cost of materials (both from Orbis). Finally, ${A}_{it}=\mathrm{exp}({c}_{o}+{\omega }_{it})$, where ${c}_{o}$ is a constant term and ${\omega }_{it}$ is firm-specific and time-varying total factor productivity, unobserved by the analyst but predictable to the firm. The level of ${\omega }_{it}$ is determined by how efficiently and intensely the inputs are utilized in production, and includes both time invariant and time varying managerial ability.

Taking logs and adding a white noise error term ${\varepsilon }_{it}$, we obtain

4 $$ln{Y}_{it}={c}_{0}+\alpha ln{E}_{it}+\rho {TI}_{it}+\delta ln{K}_{it}+\gamma ln{M}_{it}+{\omega }_{it}+{\varepsilon }_{it}$$

where we have used the approximation ln $(1+\theta {TI}_{it})\cong \theta {TI}_{it}$, and $\rho =\alpha \theta .$

There is an endogeneity problem if ${\omega }_{it}$ correlates with optimal input decisions. A classical solution to this problem has been proposed by Olley and Pakes (1996), and is based on the idea that ${\omega }_{it}$ can be eliminated from Eq. (4) by substitution if an additional equation exists that monotonically associates it with an observable variable (i.e. investment or the cost of materials). In this paper, we follow Levinsohn and Petrin (2003), and use the cost of materials *M* as the control variable required to learn about total factor productivity ${\omega }_{it}$.

Table 6 reports our estimates of Eq. (4) using the Levinsohn-Petrin method. We find that training investment per employee has a positive and statistically significant effect on log output per head (estimated coefficient: 0.112, standard error: 0.013), and that a 10 percent increase in investment in employee training—which in our data corresponds on average to 22.7 euro—raises firm productivity by 0.25 percent. We also find that a 10 percent increase in the capital stock—induced by more investment in physical capital—increases productivity by 1.53 percent.

**Table 6:** The effect of investment in training per employee (TI) on firm productivity. Production function estimates. Method: Levinsohn and Petrin. 2015–18

Variable	
Training per employee	0.112***
	(0.013)
Log employment	0.373***
	(0.010)
Log fixed assets	0.153***
	(0.012)
Log material costs	0.475***
	(0.025)
Sample size	23,663

The estimates are based on the routine “prodest” in Stata 16. Additional controls include country by year and sector fixed effects. Standard errors within parentheses. *, **, ***for statistical significance at the 10, 5 and 1 level of confidence

According to the CVTS, the average cost of an hour of training in 2015 in the EU27 Member States and the UK was 58 euro. Our estimates imply that one additional hour of training per employee would increase productivity by 0.64 percent (58/22.7 × 0.25), a relatively large effect, at least when compared to Almeida and Carneiro (2009), who estimate for Portugal that 10 additional hours of training per employee increase productivity by 0.6 to 1.3 percent.^28^ When we restrict our estimates to countries in Southern Europe, however, we find that an additional hour of training is expected to increase productivity by 0.2 percent, much closer to the estimates for Portugal.^29^

## Robustness checks

Table 7 presents the results of five different robustness checks. A potential threat to the validity of the instrument used in this paper is that, if higher quality firms systematically choose “better” banks, which have a lower non-performing loan ratio, NPL could be negatively correlated with unobserved firm quality. A negative correlation between NPL and the error term in Eq. (2) could also be due to the fact that both “better” banks and “better” firms sort into more dynamic economic areas.

**Table 7:** The effect of the financing constraints index (FC) on investment in training per employee (TI) and physical capital per employee (KI). IV estimates. Robustness checks

Variable	Training	Physical capital
*A. Adding total factor productivity and regional GDP*		
FC	− 0.000 (0.051)	− 3.106* (1.623)
F-test first stage	22.40	23.52
Sample size	28,494	27,553
*B. Excluding countries with many imputed values of leverage and cash flow*		
FC	0.063 (0.071)	− 4.281* (2.347)
F-test first stage	10.96	13.36
Sample size	15,591	15,058
*C. Excluding 2018*		
FC	0.029 (0.053)	− 3.180* (1.764)
F-test first stage	15.50	17.49
Sample size	21,558	20,876
*D. Excluding all imputed values of leverage and cash flow*		
FC	0.020 (0.049)	− 2.792 (1.828)
F-test first stage	12.40	14.86
Sample size	15,505	14,965
*E. Using the alternative index of financing constraints FC2*		
FC2	0.002 (0.135)	− 8.321*** (4.165)
F-test first stage	16.33	17.18
Sample size	28,494	27,553

All regressions include country by year and sector fixed effects, indicator variables for missing values, subsidiary and foreign status, for being hampered by business regulations, labour market regulations and lack of the right skills, the first and second lag of return on equity and real sales per employee, firm age and the log of total real assets. Standard errors (in parentheses) are clustered by firm. Bootstrapped standard errors in section e. *, **, ***for statistical significance at the 10, 5 and 1 level of confidence

To address this issue, we augment our regressions with predicted total factor productivity ${\widehat{\omega }}_{it}$, an indicator of firm quality that we obtain from our estimates of Eq. (4), and with lagged local real GDP – where the local area is identified using the NUTS 1 classification. When we do so, our results are qualitatively unchanged (see panel A of Table 7). In particular, we find that the estimated effect of the FC index on investment in training and physical capital is equal to zero (standard error: 0.051) and − 3.106 (standard error: 1.623, statistically significant at the 10 percent level of confidence) respectively.^30^

We also estimate Eq. (2) by restricting our sample to the 11 countries with a share of imputed observations below 35 percent for the cash flow to assets ratio and leverage.^31^ Although the sample size declines considerably (15,591 observations for investment in training and 15,058 observations for investment in physical capital), our estimates remain qualitatively similar to those reported in Table 5 (see panel B of Table 7), as we estimate that the effect of the FC index is equal to 0.063 (standard error: 0.070) for training investment and to − 4.281 (standard error: 2.348, statistically significant at the 10 percent level of confidence) for investment in physical capital.

Since the percentage of missing values in Orbis is higher in 2018 than in previous years, we re-estimate our empirical model on the sub-period 2015–2017 (see panel C of Table 7). We find that a one standard deviation increase in FC has no statistically significant effect on training investment (estimated coefficient: 0.029; standard deviation: 0.053) but reduces the investment in physical capital by 3.180 (standard error: 1.764, statistically significant at the 10 percent level of confidence). These effects are very similar to those reported in Table 5.

We also consider the sub-sample of firms with no imputed value for leverage and the cash flow to assets ratio. As shown in Panel D of the table, the estimated effects are similar to those in Table 5, but less precisely estimated, as one would expect given the much smaller sample size. We find that the effect of a one standard deviation increase in FC is 0.020 (standard error: 0.049) on training investment and − 2.792 (standard error: 1.828) on investment in physical capital.

Finally, we construct an alternative index of financing constraints using regression analysis, as in Lamont et al. (2001), and recognizing that these constraints could be affected also by firm size and age (see Hadlock & Pierce, 2010). The index FC2 is obtained by regressing the variable AC (self-reported financing constraints) on leverage, the cash flow to assets ratio, firm age and firm size (measured by the log of real total assets) and binary variables for missing values. We find that self-reported constraints increase with leverage (estimated coefficient: 0.029; standard error: 0.007) and decline with cash flow (estimated coefficient: − 0.132; standard error: 0.021), firm size (estimated coefficient: − 0.007; standard error: 0.001) and firm age (estimated coefficient: − 0.011; standard error: 0.002). Using these estimates, we compute the index as

$$FC2=0.029 Leverage-0.132 Cash flow-0.007 Size-0.010 Age$$

The correlation between standardized FC and FC2 is 0.523. We replicate our estimates in Table 5 by replacing FC with FC2 and obtain the results shown in panel E of Table 7, which confirm that financing constraints do not affect training investment but substantially reduce investment in physical capital.^32^

## Conclusions

Employer investment in employee training varies substantially within the European Union. Firms in Central and Eastern Europe and in Southern Europe invest much less than firms located in Northern and Western Europe. In this paper, we have investigated whether this cross-country variation can be explained at least in part by differences in the financing constraints that firms face, which may affect their investment activities. To address this question, we have matched firm survey data from EIBIS, which cover the 27 EU Member States and the UK, with administrative data on the balance sheets of firms from the Orbis database and banks’ data on financial ratios from BankScope.

We have argued that relying only on self-reported indicators may fail to consider that access to external finance is typically more problematic for firms with high leverage—measured as the debt to assets ratio—and low cash flow. Using OLS estimates, we have shown that the impact of financing constraints on training investment turns from positive to negative when we measure these constraints by considering the contribution of balance sheet information.

We have estimated the causal impact of this index on investment in training and physical capital per employee using an instrumental variable (the NPL ratio of the bank that provided loans to the firm), and found that a one standard deviation increase in the index has no statistically significant effect on training investment and large, negative and precisely estimated effects on investment in physical capital. The difference between these estimates is statistically significant.

Our finding that financing constraints do not affect training investment does not confirm previous estimates by Popov (2014), who concluded that higher constraints reduce training. There are several potential reasons why our results differ. First, we use data for a different set of countries and a different time period. Perhaps more importantly, we use a different definition of training. While Popov uses a measure of the training incidence for skilled employees, we consider training investment, or expenditure on training for all employees, skilled and unskilled, and for formal and non-formal training. We think that our measure of training investment is more encompassing of the training activities taking place within firms, as it varies with training intensity, costs and duration. We cannot exclude that firms facing stronger constraints can curb formal training programs for skilled workers without reducing total expenditure. This would happen if training for the unskilled or informal training would increase.

We have shown that training per employee and physical capital positively contribute to the productivity of firms. Our empirical results indicate that financing constraints affect productivity not because they change the human capital of employees but because they alter the accumulation of physical capital.

Our estimates point to two conclusions. First, they suggest that European firms in times of financial stress cut their investment in physical capital but do not alter their training investment significantly. Second, the presence of financing constraints does not appear to be an important candidate to explain both the perceived under-investment in training and the large differences in investment in employee training observed across EU countries.

The limited effects of financing constraints on training investment could also point to the importance of more structural factors that influence firms’ training activities. These may include dedicated policies to incentivise training by firms or penalty charges for non-training firms as well as “training cultures” within firms and countries, working to support continuity of training investment.

Finally, one aspect warranting further research is the relationship between financing constraints and training investment over time. For example, the purchase of new machinery and technology can be one of the reasons to provide training for staff and if not undertaken, training investment might suffer subsequently. This would suggest a cautious take on the current situation in Europe marked by the COVID-19 shock, which could significantly impact on overall investment activities in the short- and medium-term.

## Data Availability

The data used in this paper are from the survey EIBIS (European Investment Bank Investment Survey), and can be accessed with permission by the European Investment Bank (EIB). We are willing to provide the do files required to replicate our results, but please note that access to the data requires permission by the European Investment Bank.

## Footnotes

1 The hold-up problem refers to situations where, when the investment in training is done, workers may capture part of the benefits by threatening to leave the firm. Under-investment in training in the economy may also occur because of externalities, i.e. the investing firm does not take into account that other firms and the economy at large could benefit from the investment in training (see for instance Acemoglu and Pischke, 1999, Lynch, 1994, and Bassanini et al., 2007).

2 We define credit rationing as the case in which economic agents “…would not receive a loan even if they offered to pay a higher interest rate" (Stiglitz and Weiss, 1981, p.395).

3 Financing constraints can also affect workplace safety. See Cohn et al., 2016.

4 Kaplan and Zingales define a firm as financially constrained if the costs of external funds preclude the firm from making an investment it would have undertaken had internal funds been available. The firms they classify as unconstrained or less constrained tend to have relatively large amounts of liquid assets and net worth. See Kaplan and Zingales (1997), for further discussion.

5 Their KZ index is given by: -1.002 × (cash flow to assets) + 0.283 × (Tobin Q) + 3.139 × (debt to assets)—39.368 × (dividends to assets)—1.315 × (cash to assets).

6 The sampling methodology is described in Ipsos (2017). The sample is stratified disproportionally by country, sector and size class, and stratified proportionally by region within the country. Ipsos constructed weights to reweight the sample and make it representative of the population reported by Eurostat Structural Business Statistics (SBS). Brutscher et al. (2020), provide evidence on the representativeness of EIBIS data for the business population of interest.

7 See Kalemli-Ozcan et al. (2015), for a detailed analysis of the advantages and disadvantages of using Orbis data on firms in Europe.

8 By doing so, we avoid that our estimates are driven by outliers. We prefer this solution to winsorizing the data.

9 We always include these binary variables for missing data as additional controls in our regressions.

10 By considering only monetary outlays, this definition does not consider both the opportunity costs (i.e. foregone productivity) and the indirect costs of training (the value of the time of the workers while in training).

11 In the raw EIBIS data, before any trimming is done, average training investment per employee in 2015 is equal to 1,010.9 euro, about twice the amount reported by CVTS. The EIBIS average is driven, however, by a few large outliers – the 99 percentile being equal to 7,805 euro.

12 The share refers to continuous vocational training and therefore excludes initial training and apprenticeships.

13 Among the firms reporting actual financing constraints, about 63 percent declared that they were rejected in their application for external finance. EIBIS also asks the firms to report whether they consider that the availability of external finance is a major obstacle to investment activities, a minor obstacle, or not an obstacle at all. While the measure AC is based on the experience of the firm in the most recent application for external finance, the alternative measure relies on a general perception of the respondent (see Ferrando and Mulier, 2015). In this paper, we only use the AC measure.

14 Leverage plays a central role in standard credit risk models. See for instance Merton, 1974.

15 In EIBIS, firm age is an ordinal variable taking values ranging from 1 to 5; 1: less than 2 years; 2: 2 to 5 years; 3: 5 to 10 years; 4: 10 to 20 years; 5: more than 20 years.

16 EIBIS ask firms to report whether they consider that business regulations (e.g. licences, permits, bankruptcy) and taxation, labour market regulations, and the availability of staff with the right skills is a major obstacle to investment activities, a minor obstacle, or not an obstacle at all. We construct binary variables where firms are considered “hampered by” if they report it to be a major obstacle to investment activities.

17 Since many firms appear more than once in the sample, we cluster standard errors at the level of the firm.

18 Popov, 2014takes a similar approach and uses bank branch density.

19 A loan is considered as non-performing if it satisfies either one or both of the following criteria: (a) It is a material loan which is more than 90 days past-due; (b) The debtor is assessed as unlikely to pay its credit obligations in full without realisation of collateral, regardless of the existence of any past-due amount or of the number of days past-due.

20 The mean and standard deviation of the sum of the constant term and the residuals of the regression of the NPL ratio on country by year and sector fixed effects are equal to 6.455 and 4.134 respectively. The mean and standard deviation of the NPL ratio are instead 7.125 and 6.476 (see Table 1).

21 In contrast, a ten percent increase in real sales per head has similar effects on the two types of investment, raising TI by 1.77 percent and KI by 2.56 percent.

22 Investment in land, business buildings and infrastructure amounts to 14.7 percent of total investment, which compares to 48.8 percent for investment in machinery and equipment and to 13.3 percent for investment in software, data and IT. Detailed results are available from the authors upon request.

23 The selected instruments in this case are NPL and its interaction with the binary variable equal to 1 for physical capital and 0 for training.

24 We estimate that the effect of the FC index on log average wages per employee and log employment is 0.041 (standard error: 0.121) and 0.314 (standard error: 0.238 respectively.

25 See Booth et al. (2003), for a discussion of the role of unions in the provision of training.

26 The test regresses the residuals from an IV or 2SLS regression on all instruments. Under the null hypothesis that all instruments are uncorrelated with the error term, the test has a large-sample Chi squared(r) distribution, where *r* is the number of over-identifying restrictions.

27 See, for example, Almeida and Carneiro (2009), Konings and Vanormelingen (2015), and Martins (2021) for recent empirical evidence of the effect of training on productivity.

28 Konings and Vanormelinger, 2015, find that a 10 percent increase in the share of trained workers raises productivity by 1.7 to 3.2 percent. Dearden et al. (2000), find that increasing the proportion of trained workers in an industry by 5 percentage points leads to a 4 percent increase in value added per hour.

29 For Cyprus, Greece, Italy, Malta, Portugal and Spain the estimated effect of TI is 0.034 (standard error: 0.029).

30 Since total factor productivity is a generated regressor, inferenced is based on bootstrapped standard errors.

31 These countries are Belgium, Bulgaria, Croatia, Denmark, Finland, France, Italy, Portugal, Slovakia, Slovenia and Spain.

32 Since FC2 is a generated regressor, inference is based on bootstrapped standard errors.
